# Occupational Class Differences in Trajectories of Working Conditions in Women

**DOI:** 10.3390/ijerph14070790

**Published:** 2017-07-14

**Authors:** Simo Raittila, Ossi Rahkonen, Eero Lahelma, Juha Alho, Anne Kouvonen

**Affiliations:** 1 Faculty of Social Sciences, University of Helsinki, 00014 Helsinki, Finland; simo.raittila@helsinki.fi (S.R.); juha.alho@helsinki.fi (J.A.); 2 Department of Public Health, University of Helsinki, 00014 Helsinki, Finland; ossi.rahkonen@helsinki.fi (O.R.); eero.lahelma@helsinki.fi (E.L.); 3 SWPS University of Social Sciences and Humanities in Wroclaw, 50-001 Wroclaw, Poland; 4 Administrative Data Research Centre (Northern Ireland), Centre for Public Health, Queen’s University Belfast, BT12 6BA Belfast, UK

**Keywords:** socioeconomic differences, ageing workers, physical working conditions, job control, job demands, occupational class

## Abstract

The aim was to examine occupational class differences in trajectories of working conditions in ageing female municipal employees. Longitudinal survey data were collected among 40 to 60-year-old employees of the City of Helsinki, Finland. The 2000–2002 baseline survey (*N* = 8960, response rate 67%) was followed up in 2007 and 2012. Only those female participants who remained employed through all three phases were included (*n* = 2540). The effects of age, occupational class, and time period on physical and psychosocial working conditions were estimated using a mixed linear growth model. Physical workload decreased with age, except for manual workers, for whom there was no change. Manual workers also had less control over their work than managers and professionals, semi-professionals, or routine non-manual employees. Job control declined similarly in all occupational classes. Although occupational class differences in the levels of job demands were found, with the managers and professionals reporting the most increased demands, job demands were fairly stable and there was virtually no age or period associated linear change in them. Age trajectories in physical workload differ by occupational class, and the differences in psychosocial working conditions between occupational classes do not converge with age.

## 1. Introduction

Many developed countries are facing difficulties with their old-age dependency ratios, as the post-war baby boomer cohorts have reached retirement age [1]. To keep the experienced employees at work for as long as possible, employers and decision-makers face the challenges of catering for the needs of their older employees. To this end, it is important to know what the most crucial problems in terms of working conditions for different employee sub-groups, such as different occupational classes, are.

Work ability and employee well-being are related to working conditions, which vary by occupational class. Poor physical and psychosocial working conditions increase the risk of work disability, retirement, and early exit from work life [2,3,4,5,6]. Working conditions are not necessarily stable, but can change over time. Eurobarometer data have shown that employees in different age groups report varying levels of job demands and job control [7]; and country-specific statistics have shown increasing job insecurity and demands following the economic downturn at the end of the last decade [8].

It would be of interest to find out whether ageing and the economic cycles differently affect the working conditions among employees representing different occupational classes. To this end, it is important to differentiate whether these time-related changes affect employees of all ages simultaneously (period effect), are associated with the personal age of the participant at each period (age effect), or show similarity only in participants born temporally close to each other and who thus share life history (cohort effect) [9]. Life-course epidemiology posits that exposures in earlier life can have long-term effects on health and health risks. These exposures can either continue for a long time or have more direct effects that are only realized for the individual later in life: the risk factors can be either biological, social, or both. With continual exposure, the effects may accumulate [10]. Through this theoretical viewpoint, we expect differential working conditions to lead, in time, to differential health outcomes. One such relationship, linking long-term adverse socioeconomic exposure to coronary heart disease, poor physical functioning, and mental ill-health, has been found in the Whitehall II study [11].

Demanding psychosocial work characteristics, such as tight time limits and conflicting demands, are more commonly associated with higher occupational positions and non-manual work than lower positions and manual work, whereas a higher physical workload is associated with manual work [12,13,14].

The lack of some characteristics of work may have also negative impacts. Having autonomy over one’s work, i.e., being able to make up one’s own mind on how and when one does one’s job, has often been found to buffer against adverse symptoms associated with high workload. Robert Karasek’s [15] Job Demand–Control Model (JDC) combined the effects of job demands and job control to indicate job strain. A collaborative meta-analysis of 13 European cohort studies found support for Karasek’s model, suggesting that high job strain is associated with an increased risk of cardiovascular disease [16]. The buffering effect of autonomy is not only limited to cardiovascular health, but has also been found to affect a wider range of symptoms and behaviours [17,18,19,20].

Recent studies have shown that an increase in physical workload and a decrease in job control may deteriorate physical health functioning [21] and mental health [22]. If there was an occupational pattern among those who experience such changes, that would give support to working conditions being one mechanism through which these health differences develop and accumulate with age.

By studying the change in the working conditions among Finnish female employees, our aim was to not only strengthen the common view that different occupational classes face different adverse physical and psychosocial working conditions, but also to find out whether the class differences persisted or changed either with the age of the employees or the period of economic stagnation in Finland following the 2008 recession.

## 2. Materials and Methods

### 2.1. Data

The data were derived from the Helsinki Health Study (HHS) cohort. The baseline surveys (Phase 1) were collected in 2000, 2001, and 2002 among all of the City of Helsinki employees turning 40, 45, 50, 55, or 60 in that particular year (*n* = 8960, response rate 69%) [23]. The first follow-up in 2007 (Phase 2) received 7332 responses, and the second follow-up in 2012 (Phase 3) 6814 responses. Non-response analyses have shown only small differences in participation by socio-demographics, which were deemed not to cause major bias [23,24].

The HHS questionnaire data are available on request. Permission can be applied for by first contacting data management at the HHS (kttl-hhs@helsinki.fi). All the members of the Helsinki Health Study research team have permission to use the questionnaire data.

For the present study, we included only women because there was not enough power for similar statistical analysis for men; 80% of participants at baseline were women, which is in accordance with the gender distribution in the Finnish municipal sector in general. Working conditions among women have also been studied less.

There is not much ethnic variation in the population sampled for the current study. At the time of the baseline survey in 2000, only 1.9% of the working-age population (18 to 64 year-olds) in Finland were immigrants, and 45% of them were not working [25]. Of the employees taking part in the HHS, less than 10% spoke other languages than Finnish as their native language, with the overwhelming majority of them being a part of the Swedish-speaking language minority. While programmes to diversify the city’s workforce have led to an increase in the proportion of employees speaking other languages to 6.9% in 2014, their combined share with Swedish-speakers is still only 11.2%, and the proportion was lower during the follow-up period of this study [26].

We included only those women who remained employed throughout the follow-up period (2000–2012). Exclusions due to missing values for physical (*n* = 73) or psychosocial (*n* = 100) working conditions or for occupational class (*n* = 40) were made. In consequence, the final analytic sample included 2540 female municipal employees.

The Ethics Committees of the Department of Public Health, University of Helsinki, and the health authorities of the City of Helsinki, Finland, approved this study.

### 2.2. Variables

#### 2.2.1. Occupational Class

The participants were categorised into four hierarchical occupational classes: manual workers (*n* = 277, e.g., cleaners and kitchen workers), routine non-manual employees (*n* = 968, e.g., childminders and care workers), semi-professionals (*n* = 590, e.g., nurses and kindergarten teachers), and managers and professionals (*n* = 705, e.g., administrators, teachers, and physicians).

#### 2.2.2. Physical Workload

Physical workload was measured by a factor score calculated from 18 questions on working conditions based on an inventory developed at the Finnish Institute of Occupational Health [27]. Appendix A includes the 18 questionnaire items.

The questions loaded on three factors. The first factor was interpreted to best measure physical workload, while the two others measure desktop job characteristics and problems with the physical work environment. The items with the largest positive (>0.20) standardized scoring coefficients for the physical load factor were awkward working positions (0.70); rotation of back (0.89); repetitive movements (0.26); and heavy physical effort or lifting and carrying heavy loads (0.24). The items with the largest negative coefficients were desktop work (−0.43), using a computer mouse (−0.25), and the dustiness of the work environment (−0.20).

For the respondents with four or less missing items, the missing responses were replaced with the mode for that item in that study period. This imputation affected 414 cases.

#### 2.2.3. Job Demands and Control

Job demands were measured as an unweighted sum of five questionnaire items (Cronbach’s alpha = 0.72). The Framingham version of job control (‘decision latitude’) was calculated from nine items (Cronbach’s alpha = 0.83). [28] Appendix A includes the questionnaire items.

If the respondent had not responded to a maximum of one job demand item, or two job control items, the missing response was replaced with the mode for that item. This imputation affected 238 cases.

#### 2.2.4. Effect of Time: Birth Cohort, Age Group, Actual Age, and Period

Different models accounted for time in different ways. In the first models, only the three periods (Phases) were used to accommodate time (Model 1), but in the further models (Models 2 and 3) the period effect was controlled for by different formulations of age.

To account for birth cohort effects (e.g., differences between participants who had started their working life in different decades), the employees were categorised into four groups with 5 years intervals by birth year. Birth cohort effects were tested, but since hardly any significant effects were found, they are not reported in detail in this study.

In the reported analyses (Models 2 and 3), the actual, continuous, and time-variant ages of the employees were used. In some analyses (data not shown), the respondents were categorised into six time-variant age groups. For example, a respondent who started out aged 40 at Phase 1 was included in (grouped to) the 40 to 45 age group to start with, but would transfer from that group to the 45 to 50 group at Phase 2 and the 50 to 55 group at Phase 3. This allowed us preliminarily to test age effects that might not be linear.

### 2.3. Statistical Analysis

The physical workload factor score and the job demand and control scores that were used as dependent variables in the analyses were standardised and grand-mean centered for the modelling, since the values do not have natural interpretations. After standardisation, they can be interpreted as positive values being above the grand mean and negative values being below it. In addition, different group means can be more easily compared. The independent variables were either grouping variables or numerical variables with naturally interpretable values (age). The mean values of the dependent variables at Phases 1, 2, and 3 are reported in Table 1 and Table 2.

A range of mixed regression models were fitted using SAS’s MIXED procedure. The estimation method was maximum likelihood (ML). The first additions to the models were the period effects, which were allowed to vary from phase to phase and the intercepts for the four occupational classes. Further models also accounted for age or birth cohort effects and interactions with occupational class. ${\mathrm{SAS}}^{®}$ version 9.4 was used for all analyses (SAS Institute Inc., Cary, NC, USA).

To account for changes in occupational class and part-time work, sensitivity analyses were conducted. In the main analyses, the occupational class was assumed not to change between periods. However, in fact there was some mainly upwards occupational mobility with approximately 37.5% of manual workers transferring to other groups (mostly to routine non-manual employment), 17.8% of routine non-manual employees transferring mostly to semi-professional jobs, 13.9% of semi-professionals transferring mostly to managerial and professional jobs, and a small fraction of 7% managers and professionals transferring mostly to semi-professional status. As it is plausible that these changes affected the working conditions of the participants, we estimated the models also with a time-variant occupational class. The results from these analyses are included in the tables in Appendix B and are briefly reported in the Results and Discussion sections.

Out of the included participants, 7% worked part-time at baseline with the proportion rising to 14% in 2012. As the proportion of part-time employees was small and the effect seems to be independent of our variables of interest, these results have been only briefly discussed in Results, and not included in the tables.

For each dependent variable, the other models were compared to an intercept-only null model to approximate the fraction of variance modelled as the proportional reduction in variance (PRV) by each further model. The null model also helps answer the most basic research question about the differences, i.e., whether there is a significant difference between individuals and phases, and thus whether it makes sense to use multilevel modelling at all [29].

## 3. Results

Table 1 shows the mean scores and their 95% confidence intervals of working conditions for the different occupational classes at each phase. When comparing different phases in each occupational class, it can be seen that most of the confidence intervals overlap.

Physical workload decreased for managers and professionals (from −0.54 in Phase 1 to −0.72 in Phase 3), semi-professionals (from 0.09 in Phase 1 to −0.09 in Phase 2), and for routine non-manual employees (from 0.51 in Phase 1 to about 0.25 in later phases), whilst for manual workers it stayed roughly the same (0.63; 0.59; 0.57).

Job demands were more stable, with only one larger drop in demands for managers and professionals between 2007 and 2012 (from 0.32 to 0.15). Job control was similarly quite stable, but decreased for managers and professionals (from 0.64 in Phase 1 to 0.51 in Phase 3) and semi-professionals (from 0.28 in Phase 1 to 0.11 in Phase 3).

Table 2 shows the phase-to-phase change by age group. Physical workload decreased from phase to phase in all age groups, and was lower in the older groups. Job demands increased for the two younger groups (40 and 45), whilst they decreased for the two older age groups (50 and 55). The level of job control did not differ much by age group, since all the values were close to the average (from -0.08 to 0.15).

As displayed in Table 3, Table 4 and Table 5, there were some differences in the growth trajectories of working conditions in different occupational classes. In the total analytic sample, physical workload decreased (Table 3, Model 2), whereas job control slightly decreased (Table 5, Model 2) over the study period. Job demands mainly remained stable (Table 4).

In addition, changes in physical workload and control were associated with age, and the difference in the growth trajectories could explain most of the differences in physical workload. Changes in physical workload were also associated with phase. These findings are explored in more detail below.

In Table 3, Table 4 and Table 5, Model 0 is an unconditional null model, Model 1 accounts for occupational class and period effects, Model 2 additionally accounts for linear age effects, and Model 3 accounted for interactions of occupational class and linear age.

These tables give the random effects of each model under error variance. These are the random effects of the models that take into account individual differences. They were also used to calculate the PRV.

The *p*-values are reported to facilitate easier reading of the tables. For fixed effects, they are the *p*-values for a *t*-test. For the error variance, they are the *p*-values for a Wald test. Significance levels are as follows: * <0.05, ** <0.01, and *** <0.001.

### 3.1. Physical Workload

As Table 3 shows, the level of physical workload differed by occupational class. In the models in which the different ageing trajectories were not controlled for (Models 1 and 2), managers and professionals were at a level that is over one standard deviation lower than that of manual workers (−1.23 on a standardised scale in Model 1). Semi-professionals were over half a standard deviation (−0.66 in Model 1), and routine non-manual employees about one-fourth of a standard deviation (−0.26 in Model 1) below the manual workers’ level.

When the same coefficient was estimated for all groups (Model 2), the age effect was estimated to be negative (−0.01), but when the effect was allowed to vary by occupational class (Model 3), about half of the difference between the highest and lowest occupational classes were explained (difference −0.62, CI −1.13 to −0.11). Routine non-manual employees and semi-professionals were even estimated to have a higher physical workload than manual workers at baseline. Growing older by one year was associated with an increase in physical workload for manual workers (0.01, CI 0.00–0.02), while for the other groups ageing was associated with a decrease in physical workload. For semi-professionals and routine non-manual employees, the decrease was estimated to be even twice (−0.02) the speed of increase in manual workers.

There was a statistically significant period effect from Phase 1 to Phase 2. Physical workload decreased more than a tenth of the standard deviation (the difference between Phase 1 and Phase 2 is 0.13 in Model 3). In stratified analyses conducted separately for each occupational class (data not shown), a significant amount of decrease in physical workload was found only for routine non-manual employees, which is the largest occupational class in this sample. The statistical power for the stratified analyses was not as strong as for the main models, but the non-significant period trends were similar to the ones in Model 3. The models included in the tables managed to model a decent amount (approximately 30%) of the interpersonal variation based on occupational class, and a small amount of the time-related intrapersonal change (4.1% at the highest).

Alternative models with a time-variant occupational class managed to model somewhat more of the interpersonal variance (35.1% in Model 3). The patterns in physical workload were similar, but generally the effects were weaker, with the main effects of occupational class in Model 3 not being significant in the time-variant model (see Appendix B, Table A5). Models with part-time work as an added explaining variable (data not shown) did not show a significant effect of part-time work, nor did part-time work moderate the effect of age, period, or occupational class on physical workload.

### 3.2. Job Demands

As can be seen from Table 4, managers and professionals had almost a third (0.32, CI 0.21–0.43) of a standard deviation higher level of job demands than manual workers. Semi-professionals were estimated to be at a level of 0.15 (CI 0.03–0.26, Model 1) higher than manual workers. Routine non-manual employees had the lowest demands (−0.11, CI −0.22 to −0.01; Model 1). Model 3, which controlled for the interaction effects of age and occupational class, yielded a higher level of demands for managers and professionals (0.96, CI 0.35–1.57), almost a standard deviation higher than for the other groups.

There were no significant differences in how the job demands changed for each occupational class, except for a relative decrease for the managers and professionals. The models included in Table 4 assumed period effects to be similar for all occupational classes, but even in stratified analyses no difference in period effects were found.

The models where a categorical age variable was used (data not shown) suggested that a linear model offers a poor fit for age effects on job demands. In these models, job demands were the highest around the age of 50, and many of the age groups had statistically significantly higher demands compared to the oldest age group. The same pattern can be seen in the descriptive table (Table 2), where the age group means tend to predominantly be above the grand mean for the younger groups and below it for the older ones. Also, for the younger groups, growing older between phases would seem to heighten demands, while they decrease for older age groups. A quadratic model, for example, might offer a better fit, but might also require additional repeated measurements [29]. Furthermore, the linear models explained only a very small part of the variation in job demands over the study period and between the groups. The explained interpersonal variation was close to 7%, while the PRV for intrapersonal (Level 1) variance could be rounded to zero.

Models with a time-variant occupational class and linear age (Appendix B, Table A6) explained interpersonal variance better than the model discussed here (8.3% in Model 3), but managed worse with intrapersonal variance. Patterns were otherwise similar, but taking into account the mainly upwards social mobility, differences in demands were no longer statistically significant between routine non-manual employees and manual workers. Despite high estimated demands for managers and professionals, the effects of their occupational class and their occupational age trajectory were not statistically significantly different from the comparison group (manual workers).

Models with a time-variant independent variable for part-time work (data not shown) showed that women working part-time reported significantly lower job demands (−0.18, CI −0.26 to −0.11) than women working over 30 hours a week. A further model estimating the interaction effects of occupational class and part-time work showed significant difference in how part-time work affects routine non-manual employees compared to manual workers, with part-time work reducing job demands for manual workers (−0.33, CI −0.55 to −0.10), but not for non-manual employees (interaction: 0.29, CI 0.03–0.54). However, part-time work did not moderate the age, period, or occupational class effects.

### 3.3. Job Control

As depicted in Table 5, there were statistically significant (*p* < 0.001) occupational class differences in job control. On average, professionals and managers reported over one (1.28, CI 1.17–1.38 in Model 1) and semi-professionals almost one standard deviation (0.9, CI 0.79–1.01 in Model 1) more job control than manual workers. Routine non-manual employees had over a third (0.37, CI 0.27−0.47 in Models 1–2) of a standard deviation more job control than manual workers.

Job control was negatively (−0.01, CI −0.02–0.00) associated with age (Model 2), and this change by age did not vary by occupational class (Model 3). Different survey phases were not associated with job control when age was adjusted for (Model 1 vs. Models 2 and 3). Stratified analyses for period effects (data not shown) showed that when age was controlled for, there was a statistically significant (*p* < 0.01) increase in job control for managers and professionals between 2007 and 2012. The PRV for intrapersonal variance was from 0.8% to 1% for the models, while occupational class explained about 30% of interpersonal variance.

The patterns in models with a time-variant occupational class (Appendix B, Table A7) were similar to those shown in Table 4, but the effects were somewhat weaker. In models with a time-variant independent variable for part-time work (data not shown), the patterns stayed the same, but part-time work had in itself a significant association with job control, with part-time employees reporting less control (−0.10, CI −0.16 to −0.04). Interactions of occupational class and part-time work were not significant.

## 4. Discussion

We found clear differences in the levels of physical workload and job control between female employees representing different occupational classes. If the grand mean of the study is at the top of a bell curve, then the manual workers and routine non-manual employees are on a completely different side of the curve compared to managers, professionals, and semi-professionals. This is in line with earlier studies, in which, e.g., routine non-manual employees and manual workers have been shown to have different working conditions [14,30]. Such a difference in work characteristics is, in fact, one idea behind most occupational class categorisations.

More importantly, according to our results, the differences in physical workload and job control neither converge nor remain stable over time. We are not aware of earlier studies looking at the interaction effects of age and occupational class on working conditions. As to physical workload, with age manual workers actually tended to deviate more from the other occupational classes.

According to the dynamic version of the JDC-model, high job demands paired with high job control can over time facilitate more feelings of mastery, which in turn can inhibit the perception of strain and protect the employee from work-related stress. A less virtuous cycle is such that employees with high demands but comparatively less control accumulate strain and anxiety, which can hinder coping. When employees accumulate their “total lifetime exposure” to adverse (or protective) working conditions, they also become more vulnerable (or resilient) to the more straightforward effects of stress, such as cardiovascular disease [31]. This can also affect their likelihood of engaging in health-risk behaviours, but the evidence for this is less clear [32].

Of the working conditions examined in this study, differences in physical workload were associated most strongly with ageing. Routine non-manual employees and semi-professionals tended to report more physical load than manual workers, but this changed with age. This suggests that the effects of physical working conditions stack through time, and inequalities are likely to emerge from different paths and strains during a person’s life course.

However, in the present study, this effect—that the dynamics of the JDC-model would assume—was not found for the psychosocial working conditions: our results on job demands were not clear-cut, and job control decreased with age for ageing female municipal employees regardless of their occupational class. In this study’s population of interest, job demands did not seem to pattern by occupational class: in the group that has the highest demands (managers and professionals), high demands are combined with the protective higher control.

Physical strain stacking is supported by comparing the main analyses to the analyses where occupational class was treated as time-variant (Appendix B): these show that if the upwards mobility of manual workers is taken into account, the differences between occupational classes’ baseline physical workload becomes less clear. As the former manual workers started to inhabit routine non-manual jobs, the groups reported more similar physical workload than in models where we assumed these participants to still be in the context of their old jobs. Our view is that further studies should aim to clarify the effect upwards occupational mobility may have on experiences of physical and psychosocial working conditions.

According to our preliminary analyses, the effects of part-time work seem to be independent from occupational class and age effects. Part-time work also seems to affect people from different occupational classes differently. Because of this, the associations between part-time work, occupational class, and working conditions warrant further, dedicated research.

Our study did not control for non-work-related situations affecting experiences of working conditions, even though some studies have shown support for such an association, as women still carry more of, e.g., the care-burden at home, which can spill over to their working lives [33,34,35,36]. Job demands, especially, were not well explained by our models, and studies on them might benefit from a different approach. Further studies should aim to ascertain and explain the higher job demands experienced by middle-aged women compared to older women. Our results on job demands only suggest that with regard to how demanding their work is, ageing is not a linear experience for female municipal employees.

It is important to note that in the present sample, the semi-professional and routine non-manual classes included a large number of care workers, whose work contains physical strain. This makes the similarity to manual workers more understandable, but the difference in trajectories even more striking. A potential interpretation might be that the employees in the higher occupational classes have more control over the tasks they perform, and have the opportunity to transfer from more demanding physical tasks to less demanding ones as they age. That is, their higher control may help buffer the physical strain trajectory. This is plausible, as the City of Helsinki has a policy of finding their employees a new position if their health makes it difficult to continue in their current post [37,38]. However, earlier studies have not found support for an interaction between physical workload and job control [39]. Multivariate models and more stratified analyses, for example, should be employed to further investigate the relationship between physical working conditions, job control, and health [40].

### Strengths and Limitations

One of the main strengths of our study was its longitudinal design. The hierarchical linear modelling allowed us to better differentiate between age, period, and cohort effects. The present cohort is designed specifically for examining changes of physical and psychosocial working conditions, and employee health and well-being.

Physical workload declined in all classes from 2000 to 2007, but not from 2007 to 2012. This might be due to periodic changes in working conditions. However, this may not be concluded with the current evidence. The municipal sector employees in Finland may be better sheltered from economic downturns, as adapting to changes in the economic situation likely occurs at a slower pace. Longer follow-ups, extending over the economic down-cycle, could help clarify this.

Karasek and Theorell have noted that between-occupation variance estimates can be used to approximate how much of the variance in JDC-scores is due to work characteristics and how much of it is related to individual differences ([41], p. 80). According to our results, around a third of the variance in physical workload and job control can be explained by age and occupational class membership (Level 2 interpersonal variance). Then again, only about 7% of the interpersonal variance in job demands was explained by our models. These results are roughly in line with those from the study into between-occupation variance that Karasek and Theorell conducted with Joe Schwartz and Carl Pieper ([41], p. 335).

Furthermore, the sort of over-estimation of individual variance left unexplained—which Karasek and Theorell discuss in terms of “roughness of the occupational class categories” and “a baker is still a baker in the occupational class categories regardless of whether he or she works in an upscale French pastry boutique in Manhattan or supervises the slicing machine at a mass-production bread factory in Brooklyn” ([38], pp. 80–81)—should be less of an issue in our study. Our participants all work for the same employer, the City of Helsinki.

Municipal employees also have relatively stable jobs, and in our case, we included only those female cohort members who were employed at all phases. The inclusion of those employed at all three phases may have caused some selection bias due to employees experiencing adverse working conditions possibly opting out of the study or retiring. This shows especially in the small number of women aged 55 at baseline (*n* = 83). As the retirement age in Finland is between 63 and 68, many of those aged 55 at baseline were not included in this study, and one should be careful not to generalise our results to the oldest employees closest to retirement age. The relative stability and similarity within the cohort might mean that larger differences might be observed elsewhere. These kinds of biases would mainly cause our study to be overly optimistic in its estimations. It would be important to apply similar designs using data representative of the general working population.

Our working condition measures were based on self-reports, and we cannot ascertain with our data whether, for example, the physical workload among manual workers increased because their physical functioning was worsening or whether there were true changes in their work tasks. As period effects would likely affect employees of all ages simultaneously, we would suggest that the observed changes are more related to changes in the functioning among the ageing employees.

Our focus on women only was based partially on practical considerations, as typical to the Finnish municipal sector, there were far fewer men in most of the examined occupational classes. For example, there were only 177 men in routine non-manual employees at baseline: the number would have become even smaller as we focused on participants employed at all survey phases and made exclusions due to missing values. There are also more general reasons to not combine women and men in the analyses. First, men appear to be more vulnerable to psychosocial strain at work, and they are more sensitive to job control than women [35,42,43]. Second, women tend to hold different kind of positions—at work as well as at home—than men. For example, the most common job titles for manual workers in our study were bus and car drivers for men; compared to cleaners for women.

The results cannot be generalised to men and the private sector. In addition, as working conditions, the characteristics of job contracts, social benefits, and pension schemes differ between countries, the results cannot be generalised to dissimilar social contexts. Nevertheless, the present study provides novel evidence on occupational class differences in women’s working conditions that could possibly be replicated in different social, cultural, and legal contexts.

## 5. Conclusions

Taken together with earlier studies on the effects of changing working conditions [21,22], the present results further strengthen the view that physical workload is a key factor explaining socioeconomic differences in health and functioning. If the changes in working conditions affect health and functioning, and there are structural inequalities in who face such changes, a meaningful link can be proposed through which the effects of occupational class are transformed to poorer health and functioning during people’s working careers. This also suggests that preventive interventions in working conditions are needed, and that working conditions should not be assumed to be stable over time.

In general, employers and policy-makers should pay more attention to improving physical working conditions and increasing employee autonomy, especially for those working in manual positions. Indeed, it has been suggested that increasing control and reducing workload can help prevent premature retirement [44].

In any research including working conditions or occupational class, or both, as explanatory variables, it would be important to test the interactions between working conditions and age. When controlling just for age, only one age effect is estimated for all the occupational classes. This is unlikely to represent all trajectories, and can lead to worse fit of the models, and even to erroneous conclusions about the socioeconomic differences. This is in line with a point made by Tøres Theorell: “since the differences between age groups may be specific to the occupation group, corrections based upon the total working population may be erroneous” [31].

## Figures and Tables

**Table 1:** Mean working condition scores (95% confidence intervals) by occupational class and period.

Occupational Class		Phase 1 (Age Range: 40–55)	Phase 2 (Age Range: 45–62)	Phase 3 (Age Range: 50–67)
Managers & professionals *n* = 705 (28%)	Physical workload	−0.54 (−0.59, −0.48)	−0.65 (−0.71, −0.60)	−0.72 (−0.77, −0.66)
	Job demands	0.25 (0.19, 0.32)	0.32 (0.25, 0.39)	0.15 (0.07, 0.22)
	Job control	0.64 (0.59, 0.70)	0.56 (0.49, 0.62)	0.51 (0.45, 0.57)
Semi-professionals *n* = 590 (23%)	Physical workload	0.09 (0.01, 0.16)	−0.09 (−0.16, −0.01)	−0.20 (−0.27, −0.12)
	Job demands	0.04 (−0.03, 0.12)	0.05 (−0.03, 0.13)	0.11 (0.03, 0.18)
	Job control	0.28 (0.2, 0.35)	0.19 (0.12, 0.26)	0.11 (0.04, 0.18)
Routine non-manual employees *n* = 968 (38%)	Physical workload	0.51 (0.45, 0.57)	0.25 (0.19, 0.31)	0.24 (0.18, 0.30)
	Job demands	−0.22 (−0.28, −0.16)	−0.20 (−0.26, −0.14)	−0.16 (−0.22, −0.09)
	Job control	−0.33 (−0.39, −0.27)	−0.29 (−0.35, −0.23)	−0.37 (−0.43, −0.31)
Manual workers *n* = 277 (11%)	Physical workload	0.63 (0.54, 0.72)	0.59 (0.48, 0.69)	0.57 (0.47, 0.68)
	Job demands	−0.12 (−0.23, −0.01)	−0.09 (−0.21, 0.04)	−0.03 (−0.15, 0.10)
	Job control	−0.67 (−0.79, −0.55)	−0.65 (−0.76, −0.53)	−0.80 (−0.91, −0.68)

**Table 2:** Mean working condition scores (95% confidence intervals) by birth cohort and period.

Age at Phase 1		Phase 1 (Age Range: 40–55)	Phase 2 (Age Range: 45–62)	Phase 3 (Age Range: 50–67)
40 *n* = 802 (32%)	Physical workload	0.20 (0.14, 0.27)	−0.03 (−0.09, 0.04)	−0.09 (−0.16, −0.02)
	Job demands	−0.08 (−0.15, −0.02)	0.04 (−0.03, 0.11)	0.09 (0.02, 0.16)
	Job control	0.11 (0.05, 0.18)	0.10 (0.04, 0.17)	0.05 (−0.01, 0.12)
45 *n* = 887 (35%)	Physical workload	0.12 (0.06, 0.18)	−0.07 (−0.14, −0.01)	−0.11 (−0.18, −0.05)
	Job demands	0.01 (−0.05, 0.07)	0.04 (−0.03, 0.11)	0.05 (−0.01, 0.12)
	Job control	0.05 (−0.02, 0.12)	0.00 (−0.06, 0.07)	−0.06 (−0.12, 0.01)
50 *n* = 767 (30%)	Physical workload	0.09 (0.02, 0.16)	0.00 (−0.07, 0.07)	−0.04 (−0.11, 0.04)
	Job demands	0.03 (−0.05, 0.10)	−0.01 (−0.09, 0.06)	−0.11 (−0.18, −0.04)
	Job control	−0.04 (−0.12, 0.03)	−0.08 (−0.15, −0.01)	−0.20 (−0.27, −0.13)
55 *n* = 83 (3%)	Physical workload	−0.06 (−0.28, 0.17)	−0.29 (−0.49, −0.08)	−0.35 (−0.55, −0.14)
	Job demands	−0.04 (−0.28, 0.20)	−0.25 (−0.47, −0.03)	−0.34 (−0.56, −0.11)
	Job control	0.09 (−0.14, 0.31)	0.15 (−0.08, 0.38)	0.12 (−0.1, 0.34)

**Table 3:** Physical workload score: fixed effect estimates (95% confidence intervals) and error variance.

	Model 0	Model 1	Model 2	Model 3
**Fixed Effects**				
Intercept	0.00 (−0.03, 0.03)	0.51 *** (0.42, 0.60)	0.93 *** (0.54, 1.32)	0.18 (−0.39, 0.74)
Period				
2000		0.22 *** (0.19, 0.26)	0.14 *** (0.06, 0.22)	0.14 *** (0.06, 0.22)
2007		0.05 ** (0.02, 0.08)	0.01 (−0.04, 0.06)	0.01 (−0.04, 0.06)
2012		–	–	–
Occupational class				
Managers and professionals		−1.23 *** (−1.34, −1.13)	−1.24 *** (−1.34, −1.13)	−0.62 * (−1.13, −0.11)
Semi-professionals		−0.66 *** (−0.77, −0.56)	−0.67 *** (−0.78, −0.57)	0.35 (−0.17, 0.88)
Routine non-manual employees		−0.26 *** (−0.36, −0.16)	−0.27 *** (−0.37, −0.17)	0.65 ** (0.16, 1.14)
Manual workers		–	–	–
Age			−0.01 * (−0.01, 0.00)	0.01 (0.00, 0.02)
Interaction: Occupational class × age				
Managers and professionals				−0.01 * (−0.02, 0.00)
Semi-professionals				−0.02 *** (−0.03, −0.01)
Routine non-manual employees				−0.02 *** (−0.03, −0.01)
Manual workers				–
**Error variance**				
Intercept	0.6269 ***	0.4371 ***	0.4366 ***	0.4368 ***
Residual	0.3729 ***	0.3590 ***	0.3588 ***	0.3576 ***
**Model fit**				
Level 1 R^2^ (PRV)		0.037275	0.037812	0.04103
Level 2 R^2^ (PRV)		0.30276	0.303557	0.303238

PRV: proportional reduction in variance; Significance levels are as follows: * <0.05, ** <0.01, and *** <0.001.

**Table 4:** Job demands score: fixed effect estimates (95% confidence intervals) and error variance.

	Model 0	Model 1	Model 2	Model 3
**Fixed Effects**				
Intercept	0.00 (−0.03, 0.03)	−0.08 (−0.17, 0.02)	0.23 (−0.18, 0.64)	0.07 (−0.56, 0.70)
Period				
2000		−0.02 (−0.06, 0.02)	−0.08 (−0.16, 0.01)	−0.08 (−0.16, 0.01)
2007		0.01 (−0.03, 0.05)	−0.01 (−0.07, 0.04)	−0.01 (−0.07, 0.04)
2012		–	–	–
Occupational class				
Managers and professionals		0.32 *** (0.21, 0.43)	0.32 *** (0.21, 0.43)	0.96 ** (0.35, 1.57)
Semi-professionals		0.15 * (0.03, 0.26)	0.14 * (0.03, 0.25)	0.04 (−0.58, 0.66)
Routine non-manual employees		−0.11 * (−0.22, −0.01)	−0.12 * (−0.22, −0.01)	−0.13 (−0.71, 0.45)
Manual workers		–	–	–
Age			−0.01 (−0.01, 0.00)	0.00 (−0.01, 0.01)
Interaction: Occupational class × age				
Managers and professionals				−0.01 * (−0.02, 0.00)
Semi-professionals				0.00 (−0.01, 0.01)
Routine non-manual employees				0.00 (−0.01, 0.01)
Manual workers				–
**Error variance**				
Intercept	0.4635 ***	0.4317 ***	0.4308 ***	0.4311 ***
Residual	0.5364 ***	0.5361 ***	0.5363 ***	0.5349 ***
**Model fit**				
Level 1 R^2^ (PRV)		0.000559	0.000186	0.002796
Level 2 R^2^ (PRV)		0.068608	0.07055	0.069903

Significance levels are as follows: * <0.05, ** <0.01, and *** <0.001.

**Table 5:** Job control score: fixed effect estimates (95% confidence intervals) and error variance.

	Model 0	Model 1	Model 2	Model 3
**Fixed Effects**				
Intercept	0.00 (−0.03, 0.03)	−0.76 *** (−0.86, −0.67)	−0.15 (−0.54, 0.25)	−0.08 (−0.64, 0.48)
Period				
2000		0.10 *** (0.07, 0.14)	−0.02 (−0.1, 0.06)	−0.02 (−0.10, 0.06)
2007		0.08 *** (0.04, 0.11)	0.02 (−0.03, 0.07)	0.02 (−0.03, 0.07)
2012		–	–	–
Occupational class				
Managers & professionals		1.28 *** (1.17, 1.38)	1.27 *** (1.17, 1.38)	1.35 *** (0.84, 1.85)
Semi-professionals		0.90 *** (0.79, 1.01)	0.88 *** (0.77, 0.99)	1.10 *** (0.58, 1.62)
Routine non-manual employees		0.37 *** (0.27, 0.47)	0.37 *** (0.27, 0.47)	0.04 (−0.44, 0.53)
Manual workers		–	–	–
Age			−0.01 ** (−0.02, 0.00)	−0.01 * (−0.02, 0.00)
Interaction: Occupational class × age				
Managers & professionals				0.00 (−0.01, 0.01)
Semi-professionals				0.00 (−0.01, 0.01)
Routine non-manual employees				0.01 (0.00, 0.02)
Manual workers				–
**Error variance**				
Intercept	0.6479 ***	0.4537 ***	0.4514 ***	0.4519 ***
Residual	0.3520 ***	0.3492 ***	0.3492 ***	0.3484 ***
**Model fit**				
Level 1 R^2^ (PRV)		0.007955	0.007955	0.010227
Level 2 R^2^ (PRV)		0.299738	0.303288	0.302516

Significance levels are as follows: * <0.05, ** <0.01, and *** <0.001.

## Appendix A. Questionnaire Items for the Outcome Variables

**Table A1:** Physical Workload.

Instructions
Next, there will be listed some factors related to work and the working environment. Do they occur in your work and to what extent are they harmful to you? (Check one option on each line.)
Options:
□ Does not occur
□ Occurs, but is not harmful at all
□ Occurs and is somewhat harmful
□ Occurs and is very harmful
Items:
awkward working positions rotation of the back repetitive movements sitting standing walking working at a monitor using a computer mouse heavy physical effort or lifting and carrying noise vibration weak or disruptive lighting solvents, gasses or irritants warmth, coldness or changes in temperature dryness of air dirt dampness or wetness mold heavy loads

**Table A2:** Job demands.

Instructions:
Next you will be presented some statements about your work. Answer on each whether you completely agree, agree, disagree, or completely disagree with the statement. (Check one option on each line)
Options:
□ Completely agree
□ Agree
□ Neither agree nor disagree
□ Disagree
□ Completely disagree
Items:
My job requires working fast My job requires working very hard I am not asked to do an extensive amount of work I have enough time to get my job done I am free from conflicting demands others make.

**Table A3:** Job control.

Instructions:
How well do the next statements describe your job? (Check one option on every line.)
Options:
□ Completely agree
□ Agree
□ Neither agree nor disagree
□ Disagree
□ Completely disagree
Items:
My job allows me to make a lot of decisions on my own My work requires me to be creative My job requires that I learn new things My job includes a lot of repetitive work I have a lot of say about what happens on my job My job requires a high level of skill I get to do a variety of different things on my job I have an opportunity to develop my own special abilities On my job, I have very little freedom to decide how to do my work

## Appendix B. Time-Variant Occupational Class

**Table A4:** Occupational class by period and occupational class mobility.

Occupational Class	Phase 1 (2000–2002)	Phase 2 (2007)	Phase 3 (2012)	
Managers and professionals	705 (27.8%)	701 (29.0%)	725 (29.5%)	
Semi-professionals	590 (23.2%)	626 (25.9%)	660 (26.8%)	
Routine non-manual employees	968 (38.1%)	871 (36.1%)	896 (36.4%)	
Manual workers	277 (10.9%)	217 (9.0%)	181 (7.4%)	
	**Occupational Class at Phase 3 (2012)**			
**Occupational Class at Baseline**	Managers and professionals	Semi-professionals	Routine non-manual employees	Manual employees
Managers and professionals	632	42	7	0
Semi-professionals	52	493	29	1
Routine non-manual employees	35	119	768	18
Manual workers	6	6	92	162

**Table A5:** Physical workload score: fixed effect estimates (95% confidence intervals) and error variance.

	Model 0	Model 1	Model 2	Model 3
**Fixed Effects**				
Intercept	0.00 (−0.03, 0.03)	0.41 *** (0.33, 0.50)	0.87 *** (0.49, 1.25)	0.27 (−0.31, 0.84)
Period				
2000		0.19 *** (0.16, 0.23)	0.11 ** (0.03, 0.18)	0.10 * (0.02, 0.18)
2007		0.04 * (0.00, 0.07)	−0.00 (−0.05, 0.05)	−0.00 (−0.05, 0.04)
2012		–	–	–
Occupational class				
Managers and professionals		−1.07 *** (−1.17, −0.98)	−1.08 *** (−1.17, −0.99)	−0.95 (−1.13, 0.12)
Semi-professionals		−0.55 *** (−0.64, −0.46)	−0.56 *** (−0.65, −0.47)	0.38 (−0.16, 0.92)
Routine non-manual employees		−0.13 ** (−0.21, −0.04)	−0.13 ** (−0.22, −0.05)	0.39 (−0.13, 0.90)
Manual workers		–	–	–
Age			−0.01 * (−0.01, −0.00)	0.00 (−0.01, 0.01)
Interaction: Occupational class × age				
Managers and professionals				−0.01 * (−0.02, −0.00)
Semi-professionals				−0.02 *** (−0.03, −0.01)
Routine non-manual employees				−0.01 * (−0.02, −0.00)
Manual workers				–
**Error variance**				
Intercept	0.6269 ***	0.4082 ***	0.4073 ***	0.4066 ***
Residual	0.3729 ***	0.3628 ***	0.3627 ***	0.3621 ***
**Model fit**				
Level 1 R^2^ (PRV)		0.027085	0.027353	0.028962
Level 2 R^2^ (PRV)		0.348859	0.350295	0.351412

Significance levels are as follows: * <0.05, ** <0.01, and *** <0.001.

**Table A6:** Job demands score: fixed effect estimates (95% confidence intervals) and error variance.

	Model 0	Model 1	Model 2	Model 3
**Fixed Effects**				
Intercept	0.00 (−0.03, 0.03)	−0.16 ** (−0.25, −0.06)	0.15 (−0.26, 0.56)	0.35 (−0.31, 1.01)
Period				
2000		−0.00 (−0.05, 0.04)	−0.06 (−0.15, 0.02)	−0.06 (−0.15, 0.02)
2007		0.02 (−0.02, 0.06)	−0.01 (−0.06, 0.05)	−0.01 (−0.06, 0.05)
2012		–	–	–
Occupational class				
Managers and professionals		0.36 *** (0.26, 0.47)	0.36 *** (0.26, 0.47)	0.58 (0.05, 1.22)
Semi-professionals		0.20 *** (0.10, 0.31)	0.20 *** (0.09, 0.30)	−0.16 (−0.80, 0.48)
Routine non-manual employees		−0.01 (−0.11, 0.09)	−0.01 (−0.11, 0.08)	−0.48 (−1.10, 0.13)
Manual workers		–	–	–
Age			−0.01 (−0.01, 0.00)	−0.01 (−0.02, 0.00)
Interaction: Occupational class × age				
Managers and professionals				−0.00 (−0.02, 0.00)
Semi-professionals				0.01 (−0.01, 0.02)
Routine non-manual employees				0.01 (−0.00, 0.02)
Manual workers				–
**Error variance**				
Intercept	0.4635 ***	0.4256 ***	0.4247 ***	0.4252 ***
Residual	0.5364 ***	0.5380 ***	0.5382 ***	0.5368 ***
**Model fit**				
Level 1 R^2^ (PRV)		−0.002983	−0.003356	−0.000746
Level 2 R^2^ (PRV)		0.081769	0.083711	0.082632

Significance levels are as follows: * <0.05, ** <0.01, and *** <0.001.

**Table A7:** Job control score: fixed effect estimates (95% confidence intervals) and error variance.

	Model 0	Model 1	Model 2	Model 3
**Fixed effects**				
Intercept	0.00 (−0.03, 0.03)	−0.65 *** (−0.74, −0.57)	−0.02 (−0.42, 0.37)	−0.03 (−0.61, 0.55)
Period				
2000		0.15 *** (0.12, 0.18)	0.03 (−0.06, 0.11)	0.02 (−0.06, 0.11)
2007		0.09 *** (0.06, 0.12)	0.03 (−0.02, 0.08)	0.03 (−0.01, 0.08)
2012		–	–	–
Occupational class				
Managers and professionals		1.08 *** (0.98, 1.17)	1.07 *** (0.98, 1.17)	1.06 *** (0.53, 1.58)
Semi-professionals		0.70 *** (0.61, 0.80)	0.69 *** (0.60, 0.79)	0.90 ** (0.36, 1.43)
Routine non-manual employees		0.23 *** (0.14, 0.31)	0.22 *** (0.14, 0.31)	0.12 (−0.38, 0.63)
Manual workers		–	–	–
Age			−0.01 ** (−0.02, −0.00)	−0.01 * (−0.02, 0.00)
Interaction: Occupational class × age				
Managers and professionals				0.00 (−0.01, 0.01)
Semi-professionals				−0.00 (−0.01, 0.01)
Routine non-manual employees				0.00 (0.00, 0.01)
Manual workers				–
**Error variance**				
Intercept	0.6479 ***	0.4611 ***	0.4590 ***	0.4589 ***
Residual	0.3520 ***	0.3465 ***	0.3464 ***	0.3463 ***
**Model fit**				
Level 1 R^2^ (PRV)		0.015625	0.015909	0.016193
Level 2 R^2^ (PRV)		0.288316	0.291557	0.291711

Significance levels are as follows: * <0.05, ** <0.01, and *** <0.001.
